# Cytokine-Like 1 Regulates Cardiac Fibrosis via Modulation of TGF-β Signaling

**DOI:** 10.1371/journal.pone.0166480

**Published:** 2016-11-11

**Authors:** Jooyeon Kim, Jihwa Kim, Seung Hee Lee, Sacha V. Kepreotis, Jimeen Yoo, Jang-Soo Chun, Roger J. Hajjar, Dongtak Jeong, Woo Jin Park

**Affiliations:** 1 College of Life Sciences, Gwangju Institute of Science and Technology (GIST), Gwangju 61005, Korea; 2 The Cardiovascular Research Center, Icahn School of Medicine at Mount Sinai, New York 10029, United States of America; Albert Einstein College of Medicine, UNITED STATES

## Abstract

Cytokine-like 1 (Cytl1) is a secreted protein that is involved in diverse biological processes. A comparative modeling study indicated that Cytl1 is structurally and functionally similar to monocyte chemoattractant protein 1 (MCP-1). As MCP-1 plays an important role in cardiac fibrosis (CF) and heart failure (HF), we investigated the role of Cytl1 in a mouse model of CF and HF. Cytl1 was upregulated in the failing mouse heart. Pressure overload-induced CF was significantly attenuated in *cytl1* knock-out (KO) mice compared to that from wild-type (WT) mice. By contrast, adeno-associated virus (AAV)-mediated overexpression of *cytl1* alone led to the development of CF *in vivo*. The endothelial-mesenchymal transition (EndMT) and the transdifferentiation of fibroblasts (FBs) to myofibroblasts (MFBs) have been suggested to contribute considerably to CF. Adenovirus-mediated overexpression of *cytl1* was sufficient to induce these two critical CF-related processes *in vitro*, which were completely abrogated by co-treatment with SB-431542, an antagonist of TGF-β receptor 1. Cytl1 induced the expression of TGF-β2 both *in vivo* and *in vitro*. Antagonizing the receptor for MCP-1, C-C chemokine receptor type 2 (CCR2), with CAS 445479-97-0 did not block the pro-fibrotic activity of Cytl1 *in vitro*. Collectively, our data suggest that Cytl1 plays an essential role in CF likely through activating the TGF-β-SMAD signaling pathway. Although the receptor for Cyt1l remains to be identified, Cytl1 provides a novel platform for the development of anti-CF therapies.

## Introduction

Cardiac fibrosis (CF) is frequently associated with cardiac hypertrophy, atrial fibrillation, and ventricular arrhythmias, and heart failure (HF). It is characterized by the excessive deposition of extracellular matrix (ECM) molecules [1]. The fibrotic ECM molecules increase left ventricular (LV) stiffness and trigger various molecular signaling pathways, collectively resulting in the development of HF [2]. They also impair mechanoelectric coupling of cardiomyocytes, thus increasing the risk of arrhythmias [3]. Cardiac fibroblasts (FBs) play a central role in the development of CF. In response to various insults, FBs proliferate, migrate to the site of insults, and transdifferentiate into myofibroblasts (MFBs) that actively secrete fibrotic ECM molecules [4–6]. The formation of FBs during CF involved multiple mechanisms. For example, resident FBs are triggered to proliferate, and endothelial cells are substantially transformed to FBs via the endothelial-mesenchymal transition (EndMT).

**Fig 1:**
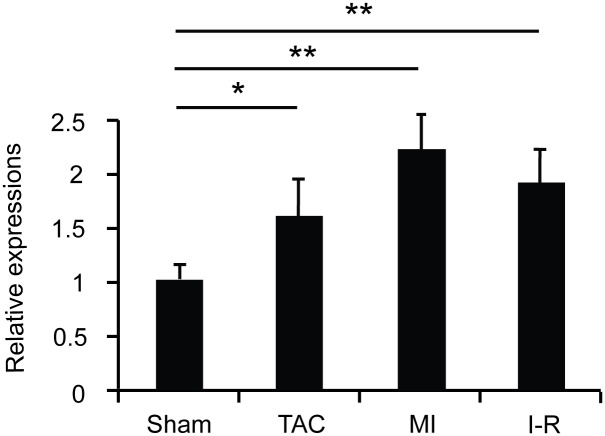
Cytl1 is upregulated under pathological conditions. Three groups of WT mice were subjected to TAC for 6 wks (TAC), ligation of coronary artery for 4 wks (MI), or ligation of coronary artery for 30 min followed by reperfusion for 24 hrs (I/R). Hearts were harvested and qRT-PCR was performed to determine the transcript levels of Cytl1. n = 3 for sham, n = 3 for TAC, n = 4 for MI, n = 4 for I/R. *p < 0.05, **p < 0.01.

Cytokine-like 1 (Cytl1) is a secreted protein first identified in CD34^+^ hematopoietic cells. It is expressed abundantly in cartilaginous tissues, including mouse inner ear and human articular cartilage[7, 8], and it functions in the chondrogenesis and cartilage homeostasis [9, 10]. Cytl1 also plays a role in the development and metastasis of neuroblastoma cells [11]. A comparative modeling study indicated that Cytl1 adopts an IL8-like chemokine fold, similar to the one present in monocyte chemoattractant protein 1 (MCP-1, also known as CCL2). Therefore, Cytl1 might be functionally related to MCP-1 that is known to be involved in the pathogenesis of CF [12–14]. We found that Cytl1 expression was highly elevated in mice with severe CF associated with pressure overload, myocardial infarction (MI), and ischemia-reperfusion (I-R) injury (Fig 1). This led us to pursue the role of Cytl1 in CF.

In this study, we found that CF was significantly attenuated in *cytl1* KO mice upon pressure overload. By contrast, adeno-associated virus (AAV)-mediated overexpression of *cytl1* resulted in the development of CF *in vivo*. Further *in vitro* experiments suggest that Cytl1 induces CF likely through activating the TGF-β-SMAD signaling pathway. Collectively, our results show that Cytl1 is a pro-fibrotic molecule in the heart. Cytl1 may serve as a therapeutic modality for CF.

## Materials and Methods

### Materials

Recombinant human TGF-β2 was purchased from PeproTech and used at a final concentration of 10 ng/ml. SB-431542 [15], an antagonist of the TGF-β receptor 1, was obtained from Sigma-Aldrich, dissolved in DMSO at a concentration of 10 mM and used at a final concentration of 10 μM. Recombinant human MCP-1 (CCL2) was purchased from R&D Systems and used at a final concentration of 20 ng/ml. CAS 445479-97-0 [16], an antagonist of CCR2, was obtained from Millipore and used at a final concentration of 6 nM.

### Animals

The mice were maintained under controlled conditions, and all animal experiments were performed with the approval of the Animal Care Committee of the Gwangju Institute of Science and Technology. The generation of Cytl1 KO mice was previously described [10]. For surgical models, male mice at 8 wks of age (23–28 g) were anesthetized with 0.5–0.7 ml of a 1x Avertin solution (a mixture of 2,2,2-tribromoethanol and tert-amyl alcohol) administered via intra-peritoneal injection. The mice were ventilated with a tidal volume of 0.1 ml and a respiratory rate of 120 breaths per minute (Harvard Apparatus).

### Transverse aortic constriction (TAC)

TAC was performed as previously described [17]. A longitudinal incision of 2 to 3 mm was made in the proximal sternum to allow visualization of the aortic arch, and the transverse aorta was ligated between the innominate and left common carotid arteries with an overlaid 27-gauge needle. The needle was then immediately removed, leaving a discrete region of constriction. Same-operated animals underwent the same surgical procedures, except that the ligature was not tied.

### Myocardial infarction (MI)

The thorax was opened under sterile conditions through a left intercostal thoracotomy and the heart was approached under direct visualization. The animal was slightly rotated to the right to enhance visualization of the left ventricle, and the left auricle was slightly retracted to fully expose the left main coronary artery system. The left anterior descending coronary artery was ligated approximately 2 mm below the tip of the normally positioned left auricle using a 7–0 silk suture. Ischemia was confirmed by discoloration of the ventricle.

### Ischemia-reperfusion (I-R)

Myocardial I-R was induced as previously described [18]. Briefly, the left anterior descending coronary artery was ligated using 7–0 silk sutured approximately 2 mm below the level of the tip of the normally positioned left auricle. Polyethylene (PE) 10 tubing with a diameter of 1 mm was placed on top of the vessel, and the suture was tied. After 30 min of occlusion, reperfusion was established by cutting the knot and removing the PE10 tubing. The chest wall was closed using 5–0 suture. Mice were sacrificed, and their hearts were removed after 24 h of reperfusion.

### Histological analysis

The paraffin-embedded heart was cross-sectioned at a thickness of 6 μm. The sections were stained with 0.1% Sirius Red solution (Sigma-Aldrich) for 1 h, and observed under an Axiophot microscope (Carl Zeiss). The fibrotic area was calculated using MetaMorph software (Molecular Devices).

### Quantitative real-time (qRT)-PCR

Total RNA was isolated with TRI Reagent (Sigma-Aldrich). Reverse transcription was performed using ImProm II reverse-transcriptase (Promega) with an oligo-dT primer. PCR was performed using an ABI PRISM Sequence Detector System 7500 (Applied Biosystems) with SYBR Green (Takara) as the fluorescent dye and ROX (Takara) as the passive reference dye. The primers used for qRT-PCR were as follows: α-SMA, 5’- ATCGT CCACC GCAAA TGC-3’ and 5’-AAGGA ACTGG AGGCG CTG-3’; Collagen 1, 5’–CGAAG GCAAC AGTCG CTTCA-3’ and 5’-GGTCT TGGTG GTTTT GTATT CCAT -3’; Cytl1, 5’- CCACC TGCTA CTCTC GGATG-3’ and 5’-CCTCG GGAAT TGGGT CTTC-3’; TGF-β2,5’-TTGCT TCAGC TCCAC AGAGA-3’ and 5’-TGGTT GTAGA GGGCA AGGAC-3’; TNF-α, 5’-CATCT TCTCA AAATT CGAGT GACAA-3’ and 5’-TGGGA GTAGA CAAGG TACAA CCC-3’;

### Western blot analysis

Heart tissue lysates were prepared by homogenization in lysis buffer (50 mM Tris-HCl, 150 mM NaCl, 0.25% Triton X-100, pH 7.4) supplemented with a protease inhibitor cocktail (Boehringer Mannheim). Approximately 50 μg of protein from each sample was separated by SDS-PAGE and transferred to a PVDF membranes (Schleicher & Schuell). The membranes were blocked with 5% non-fat milk and then incubated with primary antibodies at 4°C overnight. Antibodies for TGF-β2 and CD31 were purchased from Abcam, SMAD7 from Invitrogen, vimentin from Santa Cruz Biotechnology, Cytl1, HA, and α-SMA from Sigma-Aldrich, GAPDH, phospho-SMAD2, SMAD2/3, and VE-cadherin from Cell Signaling. The membranes were incubated with secondary antibodies conjugated to horseradish peroxidase (Jackson ImmunoResearch) and then developed with a chemiluminescent substrate (Perkin Elmer).

### Recombinant AAV production and injection

Self-complementary AAV (serotype 9) constructs were generated using the pds-AAV2-EGFP vector and the mouse *cytl1* cDNA. The recombinant AAV was produced by transfecting 293T cells as previously described [19]. The AAV particles in the cell culture media were precipitated with ammonium sulfate and purified by ultracentrifugation on an iodixanol gradient. The particles were then concentrated using a centrifugal concentrator. The AAV titer was determined by qRT-PCR and SDS-PAGE. AAV-VLP or AAV-Cytl1 (5 x 10^10^ viral genome) was injected into the tail vein of C57/BL6 mice, and the phenotype of the heart was examined after 8 wks.

### Cell culture

Human coronary artery endothelial cells (HCAEC) were cultured in EBM-2 bullet kit (Lonza). Adult primary cardiac FBs were isolated as previously described [20] and cultured in DMEM supplemented with 1% glucose (Gibco BRL).

### Recombinant adenovirus production

The AdEasy XL (Stratagene) was used to generate the recombinant adenovirus. Ad-Cytl1 was produced as previously described [17]. The viral titer was determined by the tissue culture infectious dose method. HCAECs and cardiac FBs were infected with Ad-Cytl1 for 48 h at a multiplicity of infection (moi) of 10–50.

### Fluorescent immunostaining

HCAECs and primary cardiac FBs were cultured on 12-chamber slides (Nunc). The cells were fixed with 4% paraformaldehyde for 15 min and blocked with 3–5% BSA for 30 min. The cells were then incubated with Hoechst, anti-CD31 (Santa Cruz), or anti-α-SMA antibody (Sigma-Aldrich) for 1 h 30 min, followed by incubation with FITC-conjugated secondary antibodies for 45 min. The cells were observed under a fluorescence microscope (Olympus).

### Statistics

The data were analyzed by Student’s *t*-test or one-way ANOVA, followed by the Bonferroni post-hoc test using StatView software (version 5.0, SAS). The data were expressed as mean ± SD. A *p*-value of <0.05 was considered statistically significant.

## Results

### Cytl1 is upregulated under pathological conditions

Prominent CF was observed in mice with pressure overload induced by TAC for 6 weeks, MI induced by ligation of coronary artery for 4 weeks, or I-R induced by ligation of coronary artery for 30 min followed by reperfusion for 24 h. qRT-PCR showed that the expression level of *cytl1* was significantly elevated in these failing hearts (Fig 1) suggesting a role of Cytl1 in CF.

### CF is attenuated in *cytl1* KO mice

**Fig 2:**
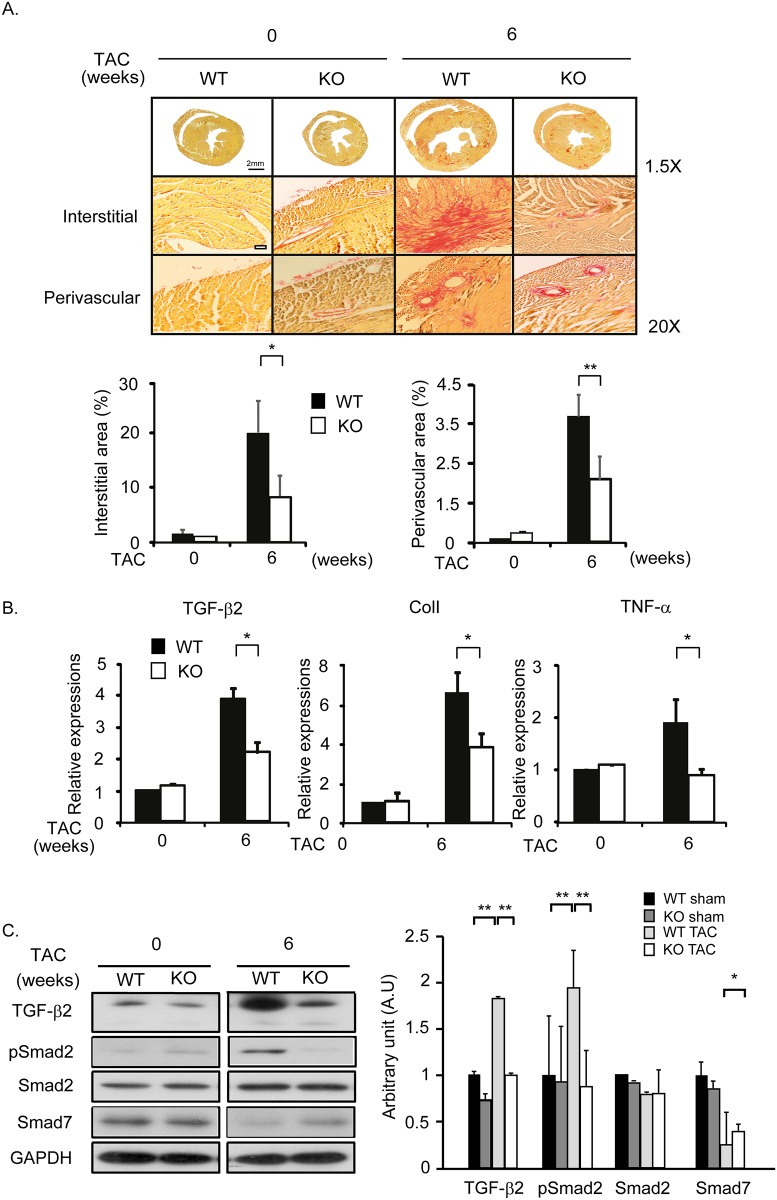
CF is attenuated in *cytl1* KO mice. WT and *cytl1* KO mice were subjected to TAC for 6 wks and the extent of fibrosis in the heart was analyzed. (A) Picrosirius staining of heart cross-sections from WT and *cytl1* KO mice subjected to TAC. Fibrotic areas in the interstitial and perivascular areas were quantified using MetaMorph software (right panels). (B) Quantification of the mRNA levels of several fibrotic markers (TGF-β2, collagen 1 and TNF-α) by qRT-PCR. (C) Activation of the TGF-β signaling pathway was investigated by western blotting. GAPDH served as the loading control. n = 3–5 for each experimental group. *p < 0.05, **p < 0.01.

Wild type (WT) and *cytl1* KO mice were subjected to TAC for 6 weeks. By Picrosirius staining, extensive CF in both interstitial and perivascular areas of the heart was observed in WT mice, but not in *cytl1* KO mice (Fig 2A). CF is typically accompanied by the increased expression of pro-fibrotic, fibrotic ECM, and pro-inflammatory markers such as TGF-β2, collagen 1, and TNF-α, respectively. qRT-PCR showed that the mRNA levels of these proteins increased significantly in WT mice; however, this fibrotic response was significantly attenuated in *cytl1* KO mice (Fig 2B). The TGF-β-SMAD signaling pathway is known to be involved in CF. This signaling pathway was activated in WT mice, as shown by the increased levels of TGF-β2 and phosphorylated SMAD2, and the decreased SMAD7 level. However, it remained unaltered in *cytl1* KO mice (Fig 2C). These data demonstrate that Cytl1 plays a critical role in CF via the TGF-β-SMAD signaling pathway.

### AAV-mediated overexpression of Cytl1 induces CF

**Fig 3:**
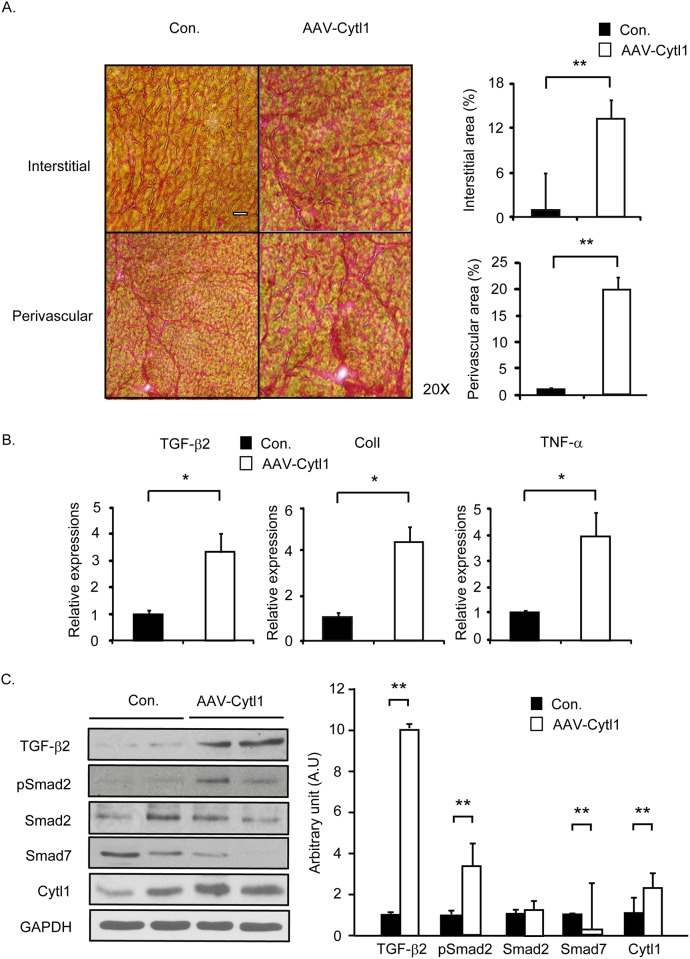
AAV-mediated overexpression of Cytl1 induces CF. Control virus or AAV-Cytl1 (5 × 10^10^ viral genome) was injected into the tail vein of WT mice, and the phenotype of the heart was examined after 8 wks. (A) Picrosirius staining of heart cross-sections from WT mice injected with control virus or AAV-Cytl. Fibrotic areas in the interstitial and perivascular areas were quantified using MetaMorph software (right panels). (B) Quantification of the mRNA levels of several fibrotic markers (TGF-β2, collagen 1 and TNF-α) by qRT-PCR. (C) Activation of the TGF-β signaling pathway was investigated by western blotting. GAPDH served as the loading control. n = 3–5 for each experimental group. *p < 0.05, **p < 0.01.

We generated a recombinant AAV (serotype 9) that expresses Cytl1 under the control of the CMV promoter (AAV-Cytl1). A two- to three-fold overexpression of Cytl1 was observed in the heart of WT mice at 8 wks after the virus (5 x 10^10^ viral genome) delivery (Fig 3C). As assessed by Picrosirius staining, the overexpression of Cytl1 significantly induced CF in both interstitial and perivascular areas of the heart (Fig 3A). qRT-PCR showed that the mRNA levels of TGF-β2, collagen 1, and TNF-α increased significantly in mice that received AAV-Cytl1 compared to those that received the control virus (Fig 3B). Western blotting results showed that Cytl1 activated the TGF-β-SMAD signaling pathway (Fig 3C). These data suggest that Cytl1 induces CF via activation of the TGF-β-SMAD signaling pathway.

### Cytl1 induces EndMT

**Fig 4:**
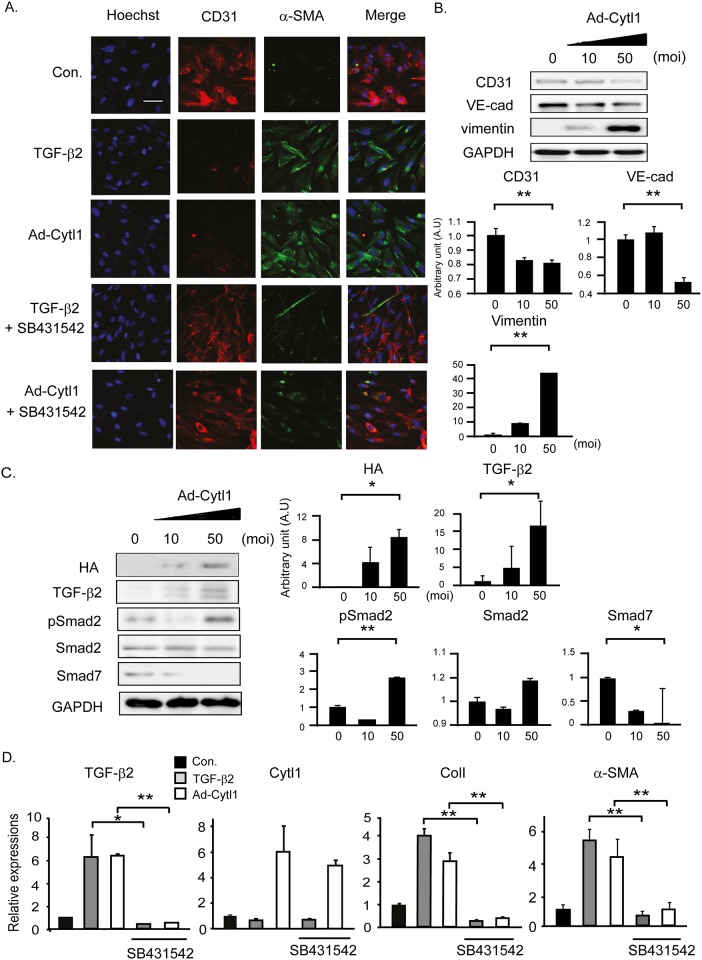
Cytl1 induces EndMT. HCAECs were treated with TGF-β2 (10 ng/ml) or Ad-Cytl1 (50 moi) for 48 h. In selected experiments, the cells were pretreated with the TGF-β receptor 1 antagonist SB431542 (10 μM). (A) The cells were immunostained with antibodies against the endothelial marker CD31 and the MFB marker α-SMA. (B) Lysates of HCAECs infected with Ad-Cytl1 were analyzed by western blotting. CD31 and VE-cadherin served as endothelial cell markers, and vimentin served as the MFB marker. (C) Activation of the TGF-β signaling pathway was investigated by western blotting. GAPDH served as the loading control. (D) Quantification of the mRNA levels of several fibrotic markers (TGF-β2, Cytl1, collagen 1 and TNF-α) by qRT-PCR. n = 3–5 for each experimental group. *p < 0.05, **p < 0.01.

Endothelial cells contribute significantly to CF through the EndMT. Thus, we tested whether Cytl1 affects this process using a recombinant adenovirus that expresses HA-tagged Cytl1 (Ad-Cytl1). TGF-β2 triggered the EndMT in human coronary artery endothelial cells (HCAECs) as shown by the downregulation of the endothelial marker CD31 and the upregulation of the mesenchymal marker, α-smooth muscle action (α-SMA). These effects of TGF-β2 were completely blocked by co-incubation with SB431542, an antagonist of the TGF-β receptor 1 (Fig 4A). These immunostaining experiments revealed that Ad-Cytl1 induced EndMT, which was further supported by the decrease of the endothelial markers CD31 and VE-cadherin and the increase of the mesenchymal marker vimentin (Fig 4B). The effects of Ad-Cytl1 were completely blocked by co-incubation with SB431542, implying that Cytl1 exerts it pro-EndMT effects via TGF-β signaling (Fig 4A). This conclusion was supported by a western blotting result, in which treatment of Ad-Cytl1 alone increased the levels of TGF-β2 and phosphorylated SMAD2, and decreased the SMAD7 level (Fig 4C). qRT-PCR showed that TGF-β2 increased the expression of TGF-β2 itself, collagen 1, and α-SMA, but not that of Cytl1, whereas Ad-Cytl1 increased the expression of Cytl1 itself, TGF-β2, collagen 1, and α-SMA. The effects of TGF-β2 and Cytl1 were blocked by co-incubation with SB431542 (Fig 3C). Collectively, these data suggest that Cytl1 induces the EndMT in HCAECs via activation of the TGF-β-SMAD signaling pathway. However, TGF-β2 does not regulate the expression of Cytl1.

### Cytl1 induces transdifferentiation of FBs to MFBs

**Fig 5:**
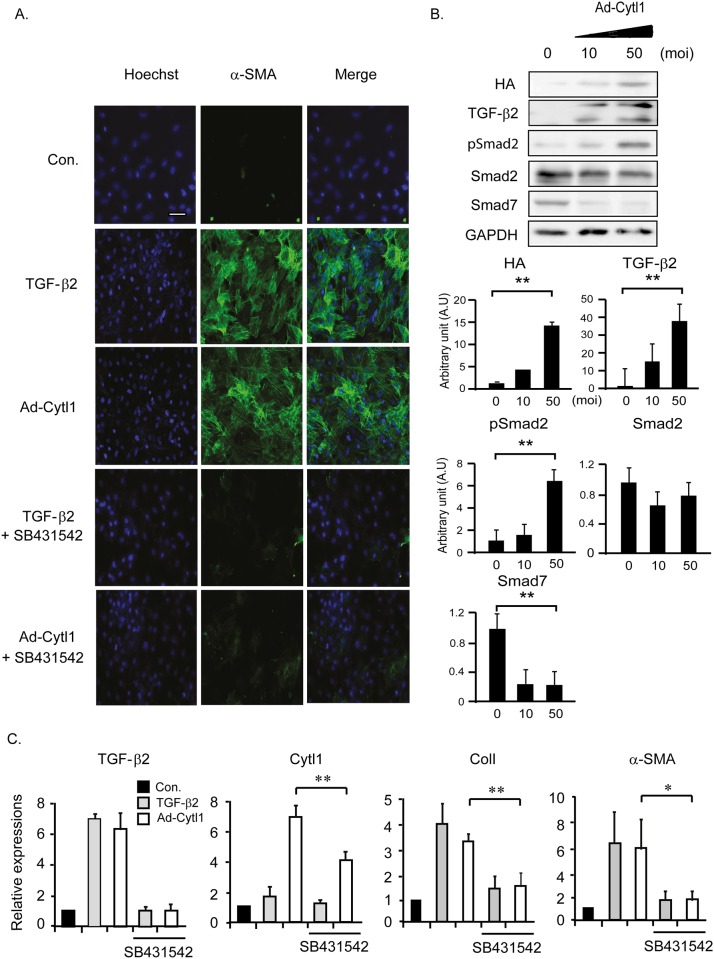
Cytl1 induces transdifferentiation of FBs to MFBs. Adult primary cardiac FBs were treated with TGF-β2 (10 ng/ml) or Ad-Cytl1 (50 moi) for 48 h. In selected experiments, the cells were pretreated with SB431542 (10 μM). (A) The cells were immunostained with an antibody against the MFB marker α-SMA. (B) Activation of the TGF-β signaling pathway in primary cardiac FBs infected with Ad-Cytl was investigated by western blotting. GAPDH served as the loading control. (C) Quantification of the mRNA levels of TGF-β2, Cytl1, the MFB marker collagen 1 and α-SMA by qRT-PCR. n = 3–5 for each experimental group. *p < 0.05, **p < 0.01.

FBs that expanded and originated from diverse sources are transdifferentiated to MFBs. The MFBs were contractile due to the increased expression of α-SMA. Both TGF-β2 and Ad-Cytl1 induced the expression of α-SMA in adult primary rat cardiac FBs, which was completely blocked by co-incubation with SB431542 (Fig 5A). Ad-Cytl1 activated the TGF-β-SMAD signaling pathway in cardiac FBs, as shown by western blotting (Fig 5B). qRT-PCR showed similar effects of TGF-β2 and Ad-Cytl1 on the mRNA levels of TGF-β2, Cytl1, collagen 1, and α-SMA in cardiac FBs similar to HCAECs (Fig 5C). These data indicate that Cytl1 induces transdifferentiation of FBs to MFBs via activation of the TGF-β-SMAD signaling pathway.

### Cytl1 functions independently of CCR2

**Fig 6:**
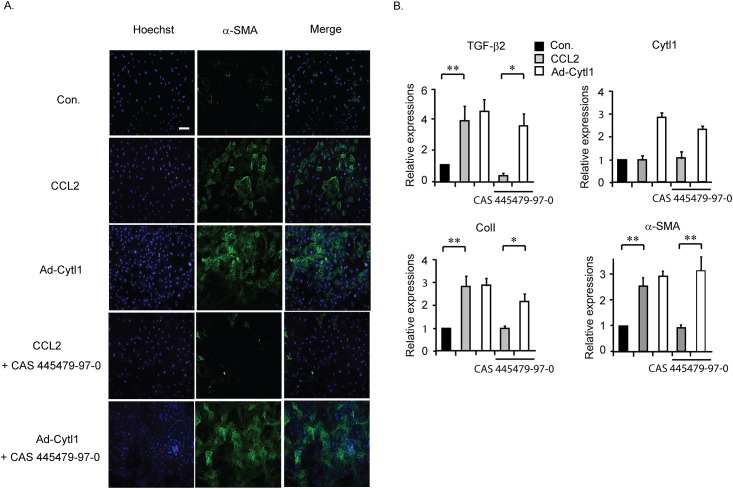
Cytl1 functions independently of CCR2. Adult primary cardiac FBs were treated with CCL2 (20 ng/ml) or Ad-Cytl1 (50 moi) for 48 h. In selected experiments, the cells were pretreated with the CCR2 antagonist CAS445679-97-0 (6 nM). (A) The cells were immunostained with an antibody against α-SMA. (B) Quantification of the mRNA levels of TGF-β2, Cytl1, collagen 1 and α-SMA by qRT-PCR. n = 3–5 for each experimental group. *p < 0.05, **p < 0.01.

A comparative modeling study indicated that Cytl1 might exert its function by activating CCR2, the MCP-1 receptor [21]. Purified MCP-1 induced the expression of β-SMA in adult rat cardiac FBs, which was completely blocked by co-incubation with CAS 445379-97-0, a CCR2 antagonist (Fig 6A). These data illustrate that CCR2 exists in cardiac FBs and is involved in CF. By contrast, the effects of Ad-Cytl1 were not affected by CAS 445379-97-0 (Fig 6A). These findings were confirmed by qRT-PCR (Fig 6B). These data suggest that Cytl1function in cardiac FBs is not mediated by CCR2.

## Discussion

Fibrosis is defined by the excessive accumulation of fibrous connective tissue (i.e., ECM components such as collagen and fibronectin) in and around damaged tissues, which can lead to permanent scarring, organ malfunction and, death [22]. CF is a hallmark of cardiac remodeling associated with various heart diseases, including hypertension, myocardial infarction, and HF. Cardiac FBs are the most prevalent cell type in the heart and they regulate cardiac function. During CF, however, they undergo proliferation and transdifferentiation to MFBs [23].

Cytl1 was first identified in CD34^+^ cells derived from bone marrow and cord blood [24]. This protein contains a putative signal peptide at its amino terminus and is secreted. With no known functions identified yet, this protein was defined as cytokine-like 1. Our group previously showed that Cytl1 is abundantly expressed in cartilage [9]. Follow-up studies using *cytl1* KO mice revealed that Cytl1 is required for the maintenance of cartilaginous homeostasis [10]. No gross morphological defects in heart, liver, spleen, and thymus were observed in *cytl1* KO mice. CD3, CD19, CD11c, and F4/80 are markers for T cells, B cells, monocytes, and macrophages, respectively. The expression levels of these markers were not significantly altered in *cytl1* KO mice as assessed by qRT-PCR, suggesting that immune responses are not affected by Cytl1 at baseline (S1 Fig). In this study, we provide compelling evidence that supports the role of Cytl1 in CF via the regulation of TGF-β-SMAD signaling. In particular, we found that Cytl1 acts directly on endothelial cells and FBs, triggering the EndMT and transdifferentiation of FBs to MFBs, the two critical processes that occur during CF.

A molecular modeling study suggested that Cytl1 contains a chemokine-fold similar to that of MCP-1 and other features necessary for signaling through the chemokine receptor CCR2 [25]. Many CXC-type chemokines, including MCP-1 (CCL2), CCL7, CCL8, and CCL13, are cognate ligands of CCR2 [26]. While preparing this manuscript, the Han group reported that Cytl1 attracts monocytes/macrophages in *in vitro* chemotaxis assays. They also showed that Cytl1 exerts this chemotactic activity through a direct binding to CCR2 [21]. These findings illustrate remarkable biochemical and functional similarities between Cytl1 and MCP-1. However, we observed that antagonizing CCR2 did not inhibit the activity of Cytl1, whereas it completely blocked the activity of MCP-1 for the transdifferentiation of FBs to MFBs. This raises the possibility that more than two different receptors for Cytl1 exist and that a yet-to-be identified receptor(s), but not CCR2, mediates the pro-fibrotic activity of Cytl1 in the heart. This hypothesis remains to be tested in future studies. In addition, we found no evidence to support a possibility that Cytl1 contributes to CF through facilitating immune responses as MCP-1 does. The expression level of markers for diverse immune cells including CD3, CD19, CD11c, and F4/80 was unaltered in *cytl1* KO mice (S2 Fig) or unaffected by Cytl1 overexpression (S3 Fig).

Previous studies suggested that CF plays a causative, but not a secondary, role in myocardial dysfunction [27]. Consistent with this hypothesis, we observed that myocardial function was significantly preserved in *cytl1* KO mice compared to that in WT mice upon pressure overload (S4 Fig) and myocardial infarction (S5 Fig). In addition, myocardial function was prominently deteriorated in mice that received AAV-Cytl1 compared to those that received control virus (S6 Fig). Collectively, these data suggest that abrogation of the Cytl1 signaling might lead to the preservation of myocardial function along with the prevention of CF. Therefore, Cytl1 might provide a novel strategy for the treatment of heart diseases such as CF and HF.

## Supporting Information

S1 Fig No significant chagnes were observed in gross morphology and the expression levels of immune cells-specific markers in *cytl1* KO mice. (A) Heart, liver, spleen, and thymus were harvested from WT and *cytl1* KO mice. (B) Whole body weight and weights of organs were measured. (C) Cytl1 is predominantly expressed in non-myocyte cells, as assessed by qRT-PCR. (D) No differences were observed in the expression level of immune cell-specific markers including CD3 (T cells), CD19 (B cells), CD11c (monocytes), and F4/80 (macrophages) in the hearts of WT and *cytl1* KO mice. WT, n = 3; *cytl1* KO, n = 3. **p < 0.01, *p < 0.05.
(TIF)

S2 Fig No significant changes were observed in the expression levels of immune cells-specific markers in *cytl1* KO mice under pressure overload. WT and *cytl1* KO mice were subjected to TAC for 6 weeks. (A) Immunohistochemistry showed no differences in the expression of CD11b/c (monocytes) and F4/80 (macrophages) between WT and i *cytl1* KO mice. (B) qRT-PCR showed no significant differences in the expression levels of CD3 (T cells), CD19 (B cells), CD11c, and F4/80. WT, n = 3; *cytl1* KO, n = 4.
(TIF)

S3 Fig Cytl1 overexpression did not affect the expression of markers for immune cells. Injection of AAV-Cytl1 through tail vein induces prominent CF. Hearts were obtained from mice injected with control or AAV-Cytl1. (A) Immunohistochemistry showed no difference in the expression of CD11b/c (a marker for monocytes) and F4/80 (a marker for macrophages) in these mice. (B) Western blotting showed no difference in the expression of CD11b/c and F4/80. AAV-Cytl1 induced about 50% increase in the expression level of Cyt1. (C) qRT-PCR showed no difference in the expression of CD3 (a marker for T cells), CD19 (a marker for B cells), CD11c, and F4/80. Con. n = 3, AAV-Cytl1 n = 3.
(TIF)

S4 Fig Contractile performance was preserved in *cytl1* KO mice after TAC. Echocardiography was performed at 1, 2, 4 and 6 wks after TAC. LVIDD, left ventricular inter-dimension at diastole; LVIDS, left ventricular inter-dimension at systole; FS, fractional shortening. *p < 0.05.
(TIF)

S5 Fig Contractile performance was preserved in *cytl1* KO mice after myocardial infarction. Echocardiography was performed at 2 and 4 wks after coronary artery ligation. LVIDD, left ventricular inter-dimension at diastole; LVIDS, left ventricular inter-dimension at systole; FS, fractional shortening. *p < 0.05, **p < 0.01.
(TIF)

S6 Fig Contractile performance deteriorated after AAV-mediated overexpression of Cytl1. Echocardiography was performed at 4 and 8 wks after tail vein injection of AAV-Cytl1. LVIDD, left ventricular inter-dimension at diastole; LVIDS, left ventricular inter-dimension at systole; FS, fractional shortening. **p < 0.01.
(TIF)
