# Supplementary figures and images for: Cytokine-Like 1 Regulates Cardiac Fibrosis via Modulation of TGF-β Signaling
Source: PLoS One. 2016 Nov 11;11(11):e0166480. doi: 10.1371/journal.pone.0166480 (PMC5105950; doi:10.1371/journal.pone.0166480)

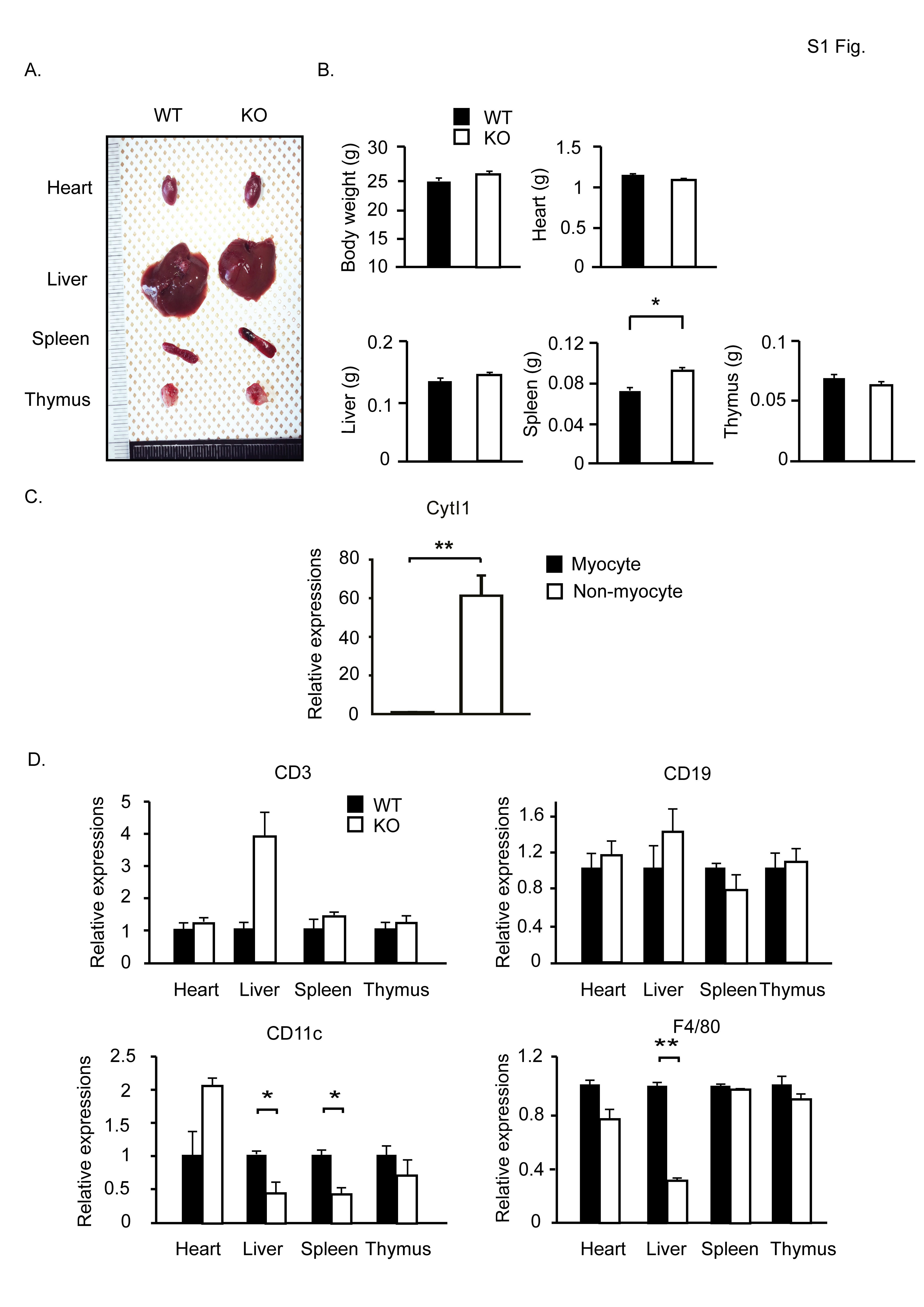

Supplement: S1 Fig — (A) Heart, liver, spleen, and thymus were harvested from WT and cytl1 KO mice. (B) Whole body weight and weights of organs were measured. (C) Cytl1 is predominantly expressed in non-myocyte cells, as assessed by qRT-PCR. (D) No differences were observed in the expression level of immune cell-specific markers including CD3 (T cells), CD19 (B cells), CD11c (monocytes), and F4/80 (macrophages) in the hearts of WT and cytl1 KO mice. WT, n = 3; cytl1 KO, n = 3. **p < 0.01, *p < 0.05. (TIF) [file pone.0166480.s001.tif]

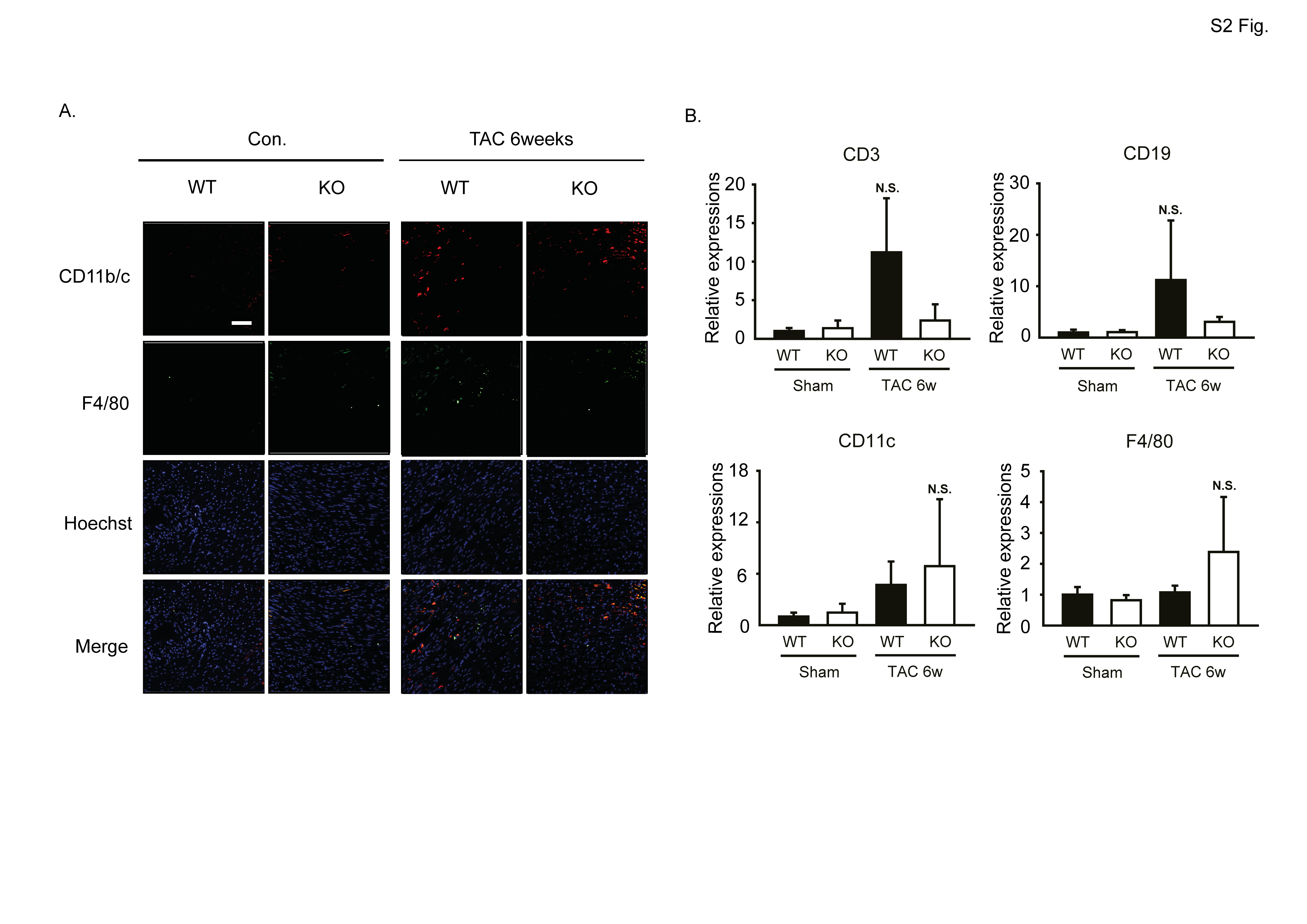

Supplement: S2 Fig — WT and cytl1 KO mice were subjected to TAC for 6 weeks. (A) Immunohistochemistry showed no differences in the expression of CD11b/c (monocytes) and F4/80 (macrophages) between WT and i cytl1 KO mice. (B) qRT-PCR showed no significant differences in the expression levels of CD3 (T cells), CD19 (B cells), CD11c, and F4/80. WT, n = 3; cytl1 KO, n = 4. (TIF) [file pone.0166480.s002.tif]

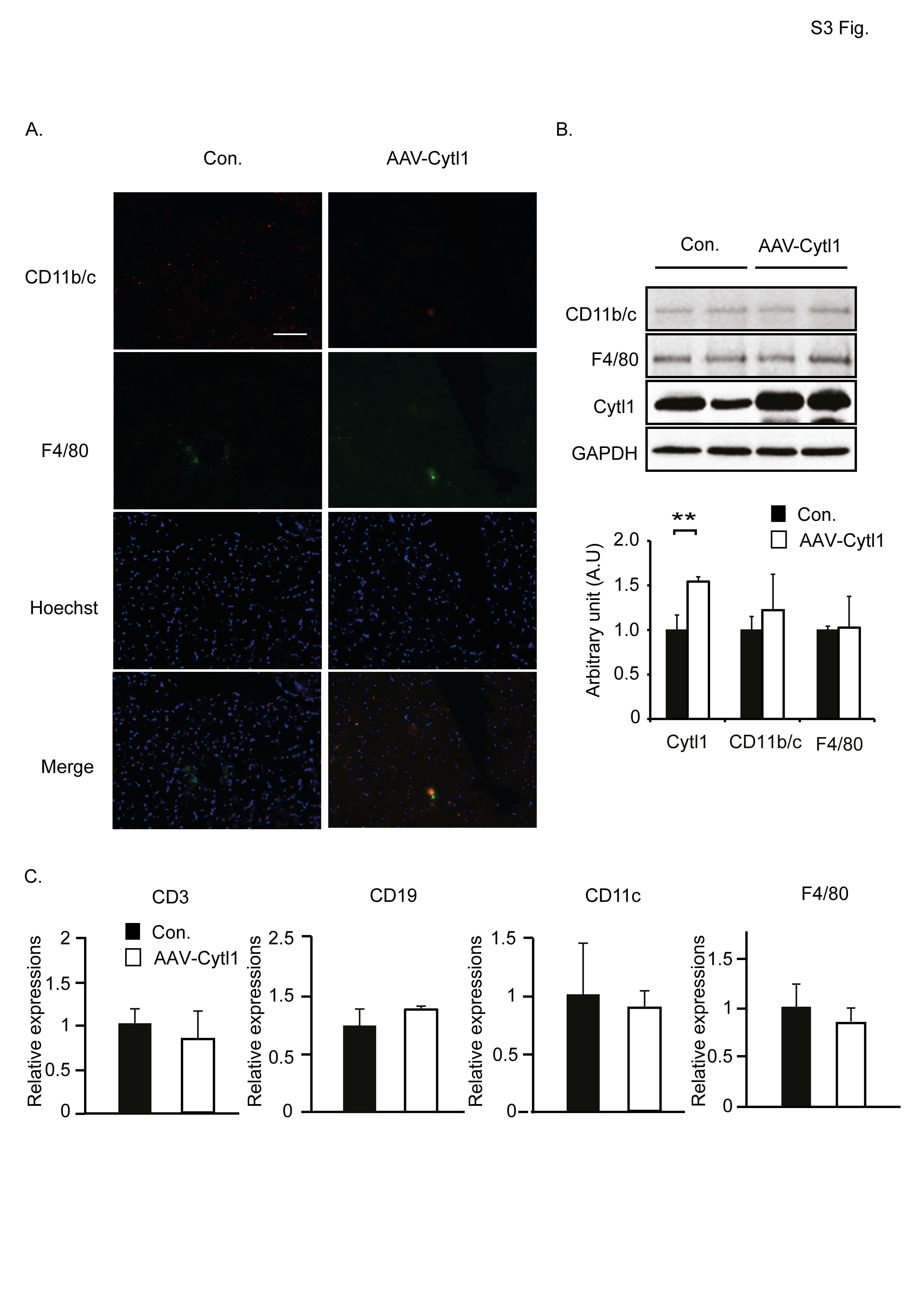

Supplement: S3 Fig — Injection of AAV-Cytl1 through tail vein induces prominent CF. Hearts were obtained from mice injected with control or AAV-Cytl1. (A) Immunohistochemistry showed no difference in the expression of CD11b/c (a marker for monocytes) and F4/80 (a marker for macrophages) in these mice. (B) Western blotting showed no difference in the expression of CD11b/c and F4/80. AAV-Cytl1 induced about 50% increase in the expression level of Cyt1. (C) qRT-PCR showed no difference in the expression of CD3 (a marker for T cells), CD19 (a marker for B cells), CD11c, and F4/80. Con. n = 3, AAV-Cytl1 n = 3. (TIF) [file pone.0166480.s003.tif]

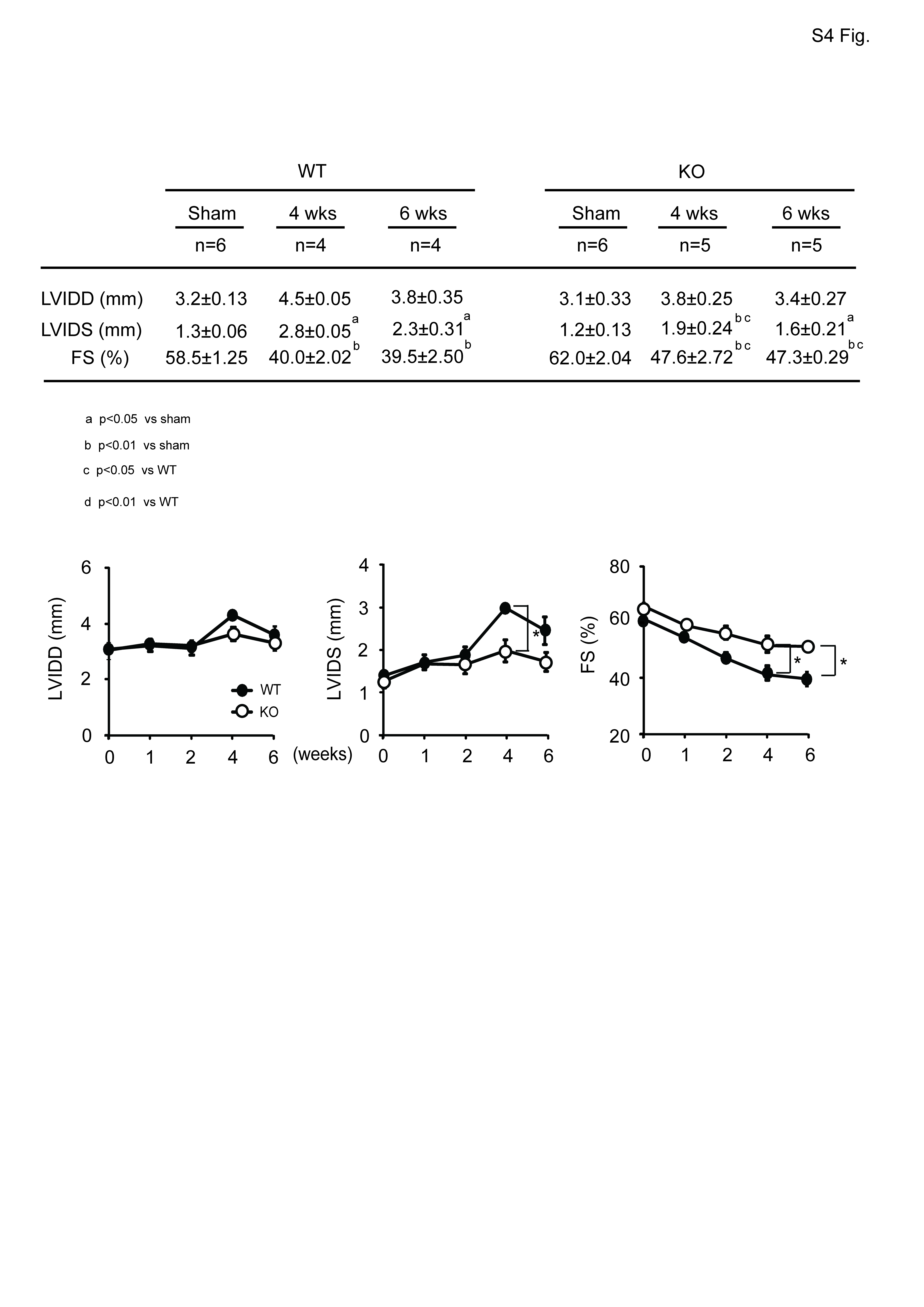

Supplement: S4 Fig — Echocardiography was performed at 1, 2, 4 and 6 wks after TAC. LVIDD, left ventricular inter-dimension at diastole; LVIDS, left ventricular inter-dimension at systole; FS, fractional shortening. *p < 0.05. (TIF) [file pone.0166480.s004.tif]

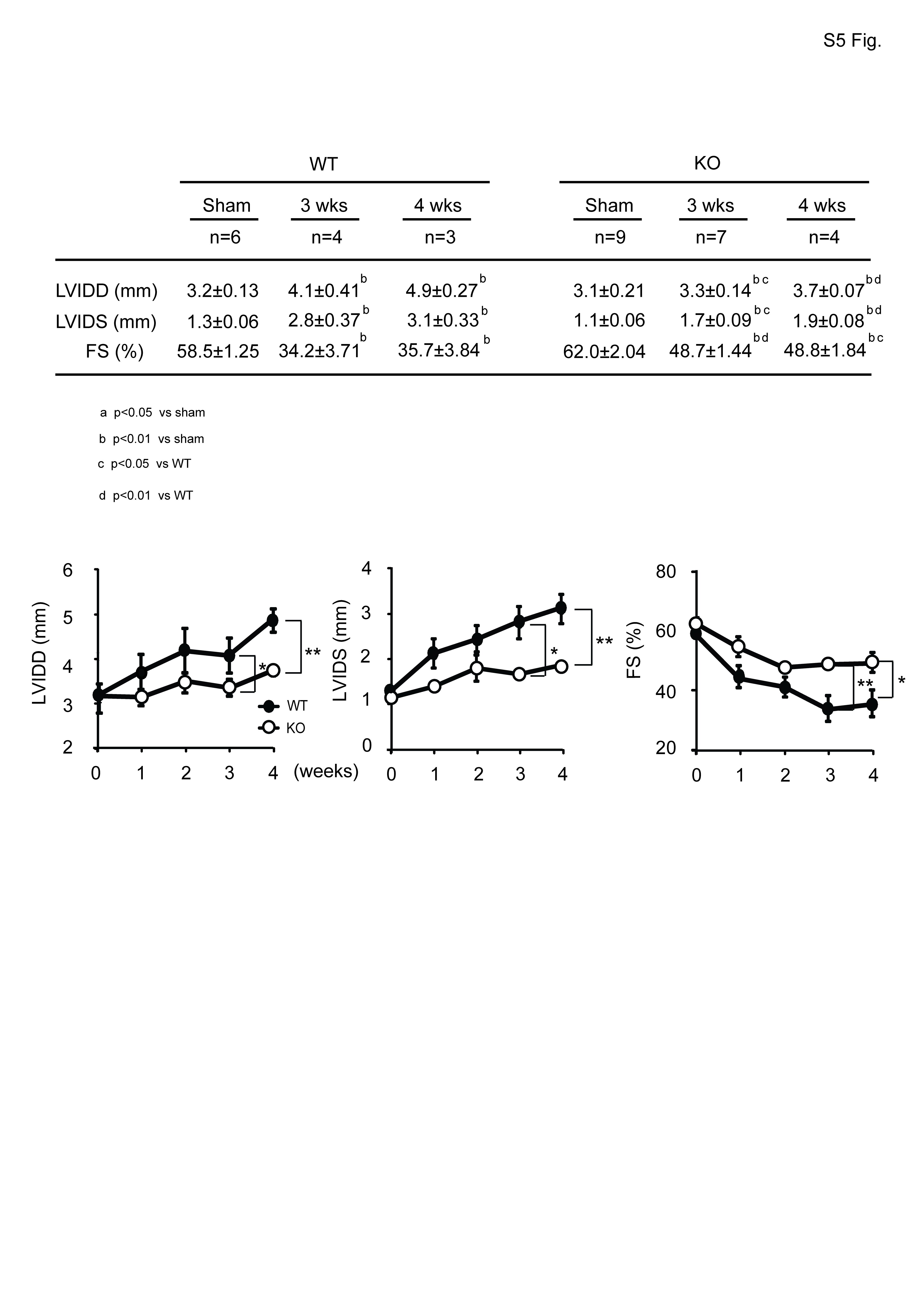

Supplement: S5 Fig — Echocardiography was performed at 2 and 4 wks after coronary artery ligation. LVIDD, left ventricular inter-dimension at diastole; LVIDS, left ventricular inter-dimension at systole; FS, fractional shortening. *p < 0.05, **p < 0.01. (TIF) [file pone.0166480.s005.tif]

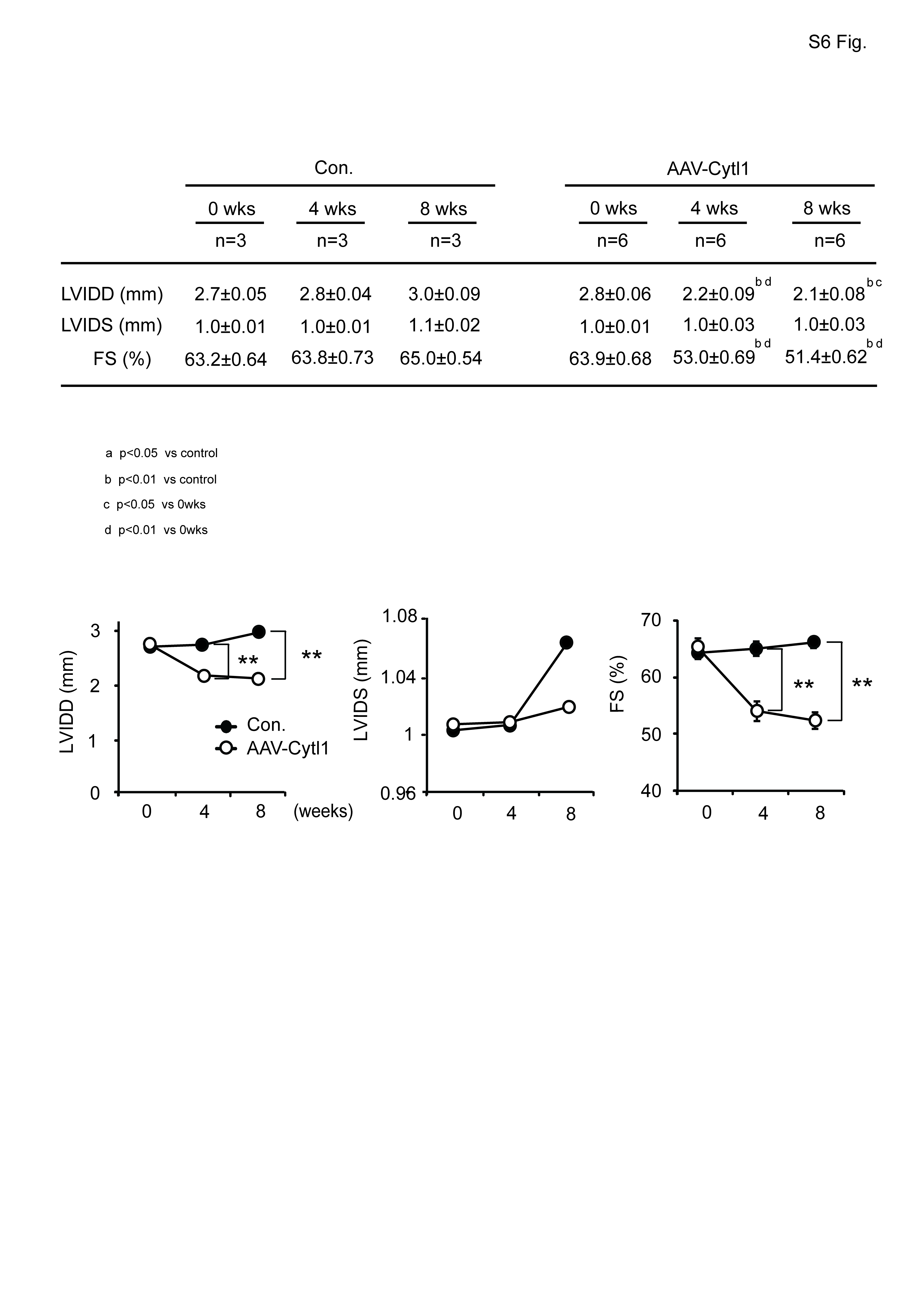

Supplement: S6 Fig — Echocardiography was performed at 4 and 8 wks after tail vein injection of AAV-Cytl1. LVIDD, left ventricular inter-dimension at diastole; LVIDS, left ventricular inter-dimension at systole; FS, fractional shortening. **p < 0.01. (TIF) [file pone.0166480.s006.tif]
